# Rapid detection of *BRCA1/2* recurrent mutations in Chinese breast and ovarian cancer patients with multiplex SNaPshot genotyping panels

**DOI:** 10.18632/oncotarget.23471

**Published:** 2017-12-20

**Authors:** Ava Kwong, John C.W. Ho, Vivian Y. Shin, Allison W. Kurian, Edmund Tai, Laura J. Esserman, Jeffery N. Weitzel, Po-Han Lin, Michael Field, Susan M. Domchek, Jessica Lo, Hextan Y.S. Ngan, Edmond S.K. Ma, Tsun L. Chan, James M. Ford

**Affiliations:** ^1^ Department of Surgery, The University of Hong Kong, Hong Kong, China; ^2^ Hong Kong Hereditary Breast Cancer Family Registry, Hong Kong, China; ^3^ Department of Surgery, Hong Kong Sanatorium & Hospital, Hong Kong, China; ^4^ Department of Medicine, Health Research & Policy and Genetics, Stanford University School of Medicine, Stanford, CA, United States; ^5^ Palo Alto Medical Foundation, Palo Alto, CA, United States; ^6^ Helen Diller Family Comprehensive Cancer Center, University of California, San Francisco, CA, United States; ^7^ Division of Clinical Cancer Genomics, City of Hope, Duarte, CA, United States; ^8^ Department of Medical Genetics, National Taiwan University Hospital, Taipei, Taiwan; ^9^ Department of Clinical Genetics, Royal North Shore Hospital, St Leonards, NSW, Australia; ^10^ Perelman School of Medicine at the University of Pennsylvania, Philadelphia, PA, United States; ^11^ Department of Obstetrics and Gynaecology, The University of Hong Kong, Hong Kong, China; ^12^ Department of Molecular Pathology, Hong Kong Sanatorium & Hospital, Hong Kong, China

**Keywords:** breast cancer, ovarian cancer, BRCA1/2, mutation, genetic testing

## Abstract

*BRCA1/2* mutations are significant risk factors for hereditary breast and ovarian cancer (HBOC), its mutation frequency in HBOC of Chinese ethnicity is around 9%, in which nearly half are recurrent mutations. In Hong Kong and China, genetic testing and counseling are not as common as in the West. To reduce the barrier of testing, a multiplex SNaPshot genotyping panel that targeted 25 Chinese *BRCA1/2* mutation hotspots was developed, and its feasibility was evaluated in a local cohort of 441 breast and 155 ovarian cancer patients. For those who tested negative, they were then subjected to full-gene testing with next-generation sequencing (NGS). *BRCA* mutation prevalence in this cohort was 8.05% and the yield of the recurrent panel was 3.52%, identifying over 40% of the mutation carriers. Moreover, from 79 Chinese breast cancer cases recruited overseas, 2 recurrent mutations and one novel *BRCA2* mutation were detected by the panel and NGS respectively. The developed genotyping panel showed to be an easy-to-perform and more affordable testing tool that can provide important contributions to improve the healthcare of Chinese women with cancer as well as family members that harbor high risk mutations for HBOC.

## INTRODUCTION

Breast cancer is the most common cause of cancer mortality in women worldwide and in Asia. In Hong Kong, breast cancer accounts for 26.6% of all newly diagnosed cancers and 10.8% of all cancer deaths in females in 2014 [1]. It is predicted that, based on the present trend, there will be an exponential increase in breast cancer incidence in Hong Kong and other Asian countries, particularly China. The fact that inhabitants of Asian countries comprise 60% of the world's population has significant implications for public health planning in the region.

Hereditary breast and ovarian cancer syndrome (HBOC) is an inherited genetic disease, of which *BRCA1* and *BRCA2* gene mutations are a prevalent cause. About 10% of the breast cancer cases in Hong Kong are inherited [2], and *BRCA* mutation carriers have a higher risk of developing breast cancer (45-65%) or ovarian cancer (11-39%) by the age of 70 than those without a *BRCA* mutation. The prevalence and risk management for *BRCA* carriers were addressed by previous pre-clinical and clinical studies, resulting in recommendations for screening and risk-reduction intervention for *BRCA* carriers and their family members. Surveillance of women with *BRCA* mutations include annual MRI breast screening from the age of 25 years alternating with mammography screening (National Comprehensive Cancer Network guidelines). Preventive alternatives include taking endocrine risk reducing drugs (tamoxifen or raloxifene) for five years, or risk-reducing mastectomy and salpingo-oophorectomy. The latter have been shown to reduce the risks of breast and ovarian cancers by 90% and 85% respectively. Risk reducing prophylactic salpingo oophorectomy is also associated with reduced overall mortality rates in *BRCA* mutation carriers [3–5]. *BRCA* mutation carriers among triple negative breast cancer (TNBC) patients are more likely to benefit from platinum-based chemotherapeutic agents [6]. A new emerging class of poly ADP-ribose polymerase (PARP) inhibitors, such as olaparib and rucaparib, also showed promising responses for ovarian cancer patients with *BRCA* defects in delaying disease progression and extending time to subsequent chemotherapy in a metastatic setting [7]. Hence, determination of *BRCA* mutation status is becoming important for clinical management with the emerging data supporting its use in therapy decisions. Also, genetic counselling and clinical interventions can be offered to family members of the affected patients.

To date, thousands of different mutations have been identified in *BRCA1* and *BRCA2* genes. However, specific *BRCA1/2* recurrent mutations were more commonly seen in different ethnic groups due to founder effect. For example, three well-characterized founder mutations (*BRCA1* c.68_69delAG, c.5266dupC and *BRCA2* c.5946delT) account for majority of the mutations identified in Ashkenazi Jews, and was reported to have a frequency of about 2.6% in the population [8–10]. In China, increasing trends in breast cancer incidence and mortality were observed, with about 270,000 new cases in 2015 [11]. Several recurrent and founder mutations have been reported in Chinese populations from different geographic locations, examples are *BRCA1* c.981_982delAT and c.5470_5477del ATTGGGCA in Eastern China [12], *BRCA2* c.3109C>T, c.7436_7805del370 and c.9097dupA in Hong Kong [13].

Although next-generation sequencing (NGS) platforms have become more popular for multi-gene panel testing [2], it is not readily accessible in most of the clinical laboratories due to limited resources, the need for bioinformaticians and data analysis of sequencing data. In the present study, we describe the development of a multiplexed, SNaPshot primer extension genotyping assay, which allows the rapid and cost-effective detection of targeted *BRCA1* and *BRCA2* mutations from breast and ovarian cancer patients in Chinese populations.

## RESULTS

### Patient and tumor characteristics

Based on the local data on *BRCA1/2* mutation spectrum and in combination with literature search, we selected 25 Chinese recurrent mutations at 13 *BRCA1* loci and 12 *BRCA2* loci (Table 1) and developed the SNaPshot genotyping panel. The panel was used to screen *BRCA* mutation in local Chinese cohorts of 441 breast cancer and 155 ovarian cancer patients, and an overseas cohort of 79 breast cancer patients. To further demonstrate the feasibility of the SNaPshot genotyping panel in Chinese populations, we also collected information of *BRCA1/2* deleterious mutations from 84 overseas Chinese carriers.

**Table 1:** List of frequently occurring *BRCA1/2* pathogenic mutations identified in Chinese breast/ovarian cancer patients

Gene	Nucleotide change	Peptide change	Exon/intron
*BRCA1*	c.470_471delCT	p.Ser157^*^	8
	c.964delG	p.Ala322Leufs^*^19	11
	c.981_982delAT	p.Cys328^*^	11
	c.2253_2254delGT	p.Met751Ilefs^*^10	11
	c.3333delA	p.Glu1112Asnfs^*^5	11
	c.3342_3345delAGAA	p.Glu1115^*^	11
	c.3916_3917delTT	p.Leu1306Aspfs^*^23	11
	c.4065_4068delTCAA	p.Asn1355Lysfs^*^10	11
	c.4148C>G	p.Ser1383^*^	12
	c.4372C>T	p.Arg1443^*^	14
	c.5406+1_5406+3delGTA	p.Asp1778Glyfs^*^27	IVS22
	c.5406+7A>G	p.Asp1778Glyfs^*^27	IVS22
	c.5470_5477del ATTGGGCA	p.Ile1824Aspfs^*^3	24
*BRCA2*	c.1832C>A	p.Ser611^*^	10
	c.2595delA	p.Glu866Lysfs^*^8	11
	c.2808_2811delACAA	p.Ala938Profs^*^21	11
	c.3109C>T	p.Gln1037^*^	11
	c.4965delC	p.Tyr1655^*^	11
	c.5164_5165delAG	p.Ser1722Tyrfs^*^4	11
	c.6591_6592delTG	p.Glu2198Asnfs^*^4	11
	c.7007G>T	p.Gly2313Alafs^*^31	13
	c.7878G>A	p.Trp2626^*^	17
	c.8068_8069delGT	p.Val2690Phefs^*^2	18
	c.9097dupA	p.Thr3033Asnfs^*^11	23
	c.9294C>G	p.Tyr3098^*^	25

A total of 441 local Chinese breast cancer probands were recruited based on the high-risk selection criteria. The mean age of diagnosis was 47.11 years with the range from 18-87 years. Of these, 84 patients (19.05%) had developed bilateral breast cancer. Majority of the tumors (60.19%) were invasive ductal carcinoma (IDC, n=316) while 15.81% were ductal carcinoma *in situ* (DCIS, n=83). The histology of the tumors were shown in Table 2, 332 (63.24%) were ER+, 44 (8.38%) were HER2+ and 59 (11.24%) were triple-negative.

**Table 2:** Characteristics of 441 local Chinese breast cancer patients screened for *BRCA1/2* mutations

	441 tested probands		35 mutation carriers	
**Mean age at diagnosis**		47.11 y		42.36 y
**Age range**		18 – 87 y		22 – 70 y
**Age at breast cancer diagnosis**	**N**	**Proportion**	**N**	**Proportion**
Below 30	14	3.17%	3	8.57%
30-39	104	23.58%	12	34.29%
40-49	173	39.23%	13	37.14%
50 or above	150	34.01%	7	20.00%
**Bilateral cases**	84	19.05%	12	34.29%
**Histology (Tumors ^a^)**	**525**	**100%**	**47**	**100%**
Ductal	316	60.19%	30	63.83%
Lobular	18	3.43%	0	-
Medullary	7	1.33%	1	2.13%
Ductal + Lobular	2	0.38%	0	-
Ductal + Medullary	1	0.19%	0	-
DCIS	83	15.81%	8	17.02%
Others	27	5.14%	1	2.13%
Unclassified	71	13.52%	7	14.89%
**Molecular subtypes (Tumors ^a^)**	**525**	**100%**	**47**	**100%**
ER+	332	63.24%	25	53.19%
ER-	11	2.10%	1	2.13%
HER2+	44	8.38%	2	4.26%
TNBC	59	11.24%	9	19.15%
Unknown	79	15.05%	10	21.28%
**Have both breast and ovarian cancer**				
Yes	10	2.27%	3	8.57%
No	431	97.73%	32	91.43%
**Family history of breast cancer (first- and second-degree relatives)**				
Yes	195	44.22%	21	60.00%
No	246	55.78%	14	40.00%

^a^ Numbers include tumors from patients with bilateral cancers.

In the unselected cohort of 155 ovarian cancer probands (Table 3), the mean age of diagnosis was 44.74 years with the range from 9-85 years, of whom 139 probands had ovarian cancers (94.56%), others had fallopian tube cancer (1.36%) and peritoneal cancer (2.04%). The vast majority (n=138, 89.03%) of patient population had epithelial ovarian tumors, in which the most common histological subtype was endometrioid carcinoma (n=50, 32.26%), followed by serous (n=36, 23.23%) and clear cell subtypes (n=24, 15.48%).

**Table 3:** Characteristics of 155 local Chinese ovarian cancer patients screened for *BRCA1/2* mutations

	155 tested probands		13 mutation carriers	
**Mean age at diagnosis**		44.74 y		53.62 y
**Age range**		9 – 85 y		37 – 85 y
**Age at ovarian cancer diagnosis**	**N**	**Proportion**	**N**	**Proportion**
Below 30	18	11.61%	0	-
30-39	20	12.90%	1	7.69%
40-49	68	43.87%	4	30.77%
50 or above	49	31.61%	8	61.54%
**Histology**				
Epithelial	138	89.03%	13	100%
Serous	36	23.23%	8	61.54%
Clear cell	24	15.48%	1	7.69%
Endometroid	50	32.26%	2	15.38%
Mucinous	16	10.32%	0	-
Mixed	12	7.74%	2	15.38%
Stromal	4	2.58%	0	-
Germ Cell	4	2.58%	0	-
Others	3	1.94%	0	-
Unclassified	6	3.87%	0	-
**Have both breast and ovarian cancer**				
Yes	9	5.81%	3	23.08%
No	146	94.19%	10	76.92%
**Family history of breast or ovarian cancer (first- and second-degree relatives)**				
Yes	38	24.52%	7	53.85%
No	117	75.48%	6	46.15%

### *BRCA1/2* mutation status in local Chinese cohorts

**Figure 1:**
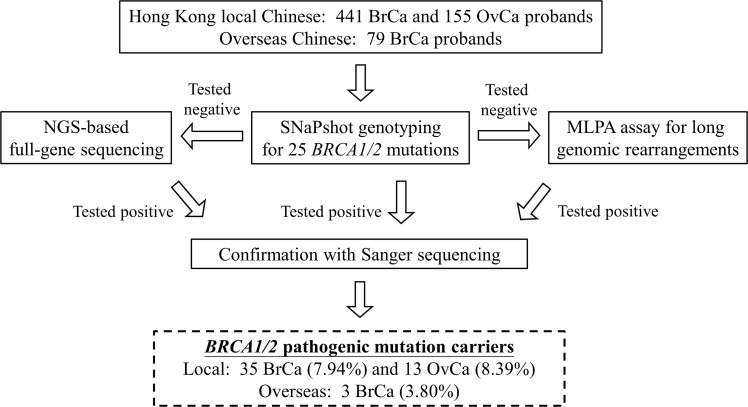
Workflow of *BRCA1/2* mutation screening of 596 breast (BrCa) and ovarian cancer (OvCa) patients (local cohort) and 79 overseas Chinese breast cancer patients

All the probands were subjected to *BRCA1/2* mutational screening with the SNaPshot genotyping panel. For those who tested negative, they were further assayed for *BRCA1*/*2* full-gene sequencing by amplicon-based NGS and large genomic rearrangements by multiplex ligation-dependent probe amplification (MLPA) (Figure 1). The prevalence of *BRCA1/2* pathogenic mutations among high-risk breast cancer patients was 7.94% (35/441). Of these, 4 *BRCA1* and 11 *BRCA2* mutation carriers were identified with the SNaPshot genotyping panel, showing a detection rate of 3.40% (15/441) among this cohort. Additionally, 5 *BRCA1* and 15 *BRCA2* mutations were identified by NGS/MLPA analysis. A great proportion of these *BRCA* carriers had family history of breast cancer (60%) compared with the non-carriers (40%) (p=0.013) (Table 2). There was a statistical difference regarding young onset ≤40 years (p=0.018) but not those ≤45 years (p=0.068) between the carriers and non-carriers.

Among the ovarian cancer patients, *BRCA* mutations were identified in 8.39% (13/155) of the patients, including 9 *BRCA1* and 4 *BRCA2*. Of these, the SNaPshot genotyping panel identified 5 *BRCA1* and 1 *BRCA2* carriers, showing a detection rate of 3.87% (6/155) among the cohort. Thirteen mutation carriers had a mean age at diagnosis of 53.6 years (range 37-85) and all had epithelial carcinoma, in which higher proportions were serous tumors (Table 3). Family history of breast/ovarian cancers is also a risk factor for detecting *BRCA* mutation between carriers (54.85%) and non-carriers (21.83%, p=0.017).

Overall, the prevalence of *BRCA1/2* mutations in this highly selected local Chinese cohort was 8.05% (48/596) and the yield of the SNaPshot genotyping panel was 3.52% (21/596), indicating that nearly half of the mutations were recurrent mutations. *BRCA1/2* mutations identified in this local Chinese HBOC cohort were listed in Table 4. Interestingly, we identified 3 *BRCA1* mutations (c.4046_4047delinsA, c.212+3A>G and c.5335delC) by NGS, which were novel to our Chinese *BRCA1/2* mutation spectrum and were only seen in ovarian cancer cases, but not in the breast cancer cases in the present cohort.

**Table 4:** *BRCA1/2* mutations identified in Chinese patients with hereditary breast and ovarian cancer syndrome

Gene	No of cases	Mutation (HGVS)	Amino acid change	Gender	Cancer type	Family history	SNaPshot panel	NGS/MLPA
*BRCA1*								
1	1	c.212+3A>G	p.Cys64^*^	F	Ovarian	No	-	NGS
2	1	c.220C>T	p.Gln74^*^	F	Breast	No	-	NGS
3	3	c.964delG	p.Ala322Leufs^*^19	F	Breast	Yes	Yes	-
				F	Ovarian	Yes	Yes	-
				F	Breast & Ovarian	Yes	Yes	-
4	2	c.2635G>T	p.Glu879^*^	F	Breast	No	-	NGS
				F	Ovarian	Yes	-	NGS
5	1	c.3333delA	p.Glu1112Asnfs^*^5	F	Ovarian	No	Yes	-
6	1	c.3342_3345delAGAA	p.Glu1115^*^	F	Breast & Ovarian	No	Yes	-
7	1	c.3756_3759delGTCT	p.Ser1253Argfs^*^10	F	Breast	No	-	NGS
8	1	c.4046_4047delinsA	p.Thr1349Lysfs^*^17	F	Ovarian	Yes	-	NGS
9	3	c.4372C>T	p.Gln1458^*^	F	Breast & Ovarian	Yes	Yes	-
				F	Breast	No	Yes	-
				F	Breast	Yes	Yes	
10	1	c.5193+1G>A	p.Trp1718Ser fs^*^2	F	Breast	Yes	-	NGS
11	1	c.5335delC	p.Gln1779Asnfs^*^14	F	Ovarian	No	-	NGS
12	1	c.5406+1_5406+3delGTA	p.Asp1778Glyfs^*^27	F	Ovarian	Yes	Yes	-
13	1	r.5194_5406del	p.His1732_Thr1802del	F	Breast & Ovarian	No	-	MLPA
*BRCA2*								
1	1	c.470_474delAGTCA	p.Lys157Serfs^*^24	F	Breast	Yes	-	NGS
2	1	c.1244delA	p.His415Leufs^*^15	F	Breast	Yes	-	NGS
3	1	c.1688G>A	p.Trp563^*^	F	Breast	Yes	-	NGS
4	1	c.1855C>T	p.Gln619^*^	F	Breast	Yes	-	NGS
5	1	c.2595delA	p.Glu866Lysfs^*^8	F	Breast	Yes	Yes	-
6	2	c.2808_2811delACAA	p.Ala938Profs^*^21	F	Breast	Yes	Yes	-
				F	Breast	Yes	Yes	-
7	7	c.3109C>T	p.Gln1037^*^	F	Breast	No	Yes	-
				F	Breast	No	Yes	-
				M	Breast	Yes	Yes	-
				F	Breast	Yes	Yes	-
				F	Breast	Yes	Yes	-
				F	Breast	Yes	Yes	-
				F	Ovarian	No	Yes	-
8	1	c.3760G>T	p.Glu1254^*^	F	Breast	Yes	-	NGS
9	1	c.4440T>G	p.Tyr1480^*^	F	Breast	Yes	-	NGS
10	1	c.5722_5723delCT	p.Leu1908Argfs^*^2	F	Breast	No	-	NGS
11	1	c.5851_5854delAGTT	p.Ser1951Trpfs^*^11	F	Breast & Ovarian	Yes	-	NGS
12	1	c.5951_5952delAA	p.Lys1984Ilefs^*^18	F	Breast	No	-	NGS
13	3	c.7133C>G	p.Ser2378^*^	F	Breast	Yes	-	NGS
				F	Breast	No	-	NGS
				F	Ovarian	No	-	NGS
14	1	c.7558C>T	p.Arg2520^*^	F	Breast	Yes	-	NGS
15	1	c.7999delA	p.Ser2776Alafs^*^6	F	Breast & Ovarian	Yes	-	NGS
16	1	c.8331+2T>C	r.7826_8331del506	F	Breast	Yes	-	NGS
17	1	c.8488-9T>G	p.Trp2830Tyrfs^*^36	F	Breast	Yes	-	NGS
18	2	c.9294C>G	p.Tyr3098^*^	F	Breast	Yes	Yes	-
				F	Breast	No	Yes	-
19	1	c.9409dupA	p.Thr3137Asnfs^*^13	F	Breast	No	-	NGS
20	1	c.10150C>T	p.Arg3384^*^	F	Ovarian	Yes	-	NGS

All the *BRCA1/2* mutations detected by SNaPshot genotyping panel and NGS were confirmed by independent Sanger sequencing, and the results were consistent. In addition, no false negative from the SNaPshot genotyping panel was seen, implicating the accuracy and feasibility of the panel.

### *BRCA1/2* mutation in overseas Chinese breast cancer patients

In the second part of this study, we collected 79 blood samples from Chinese breast cancer probands residing in overseas countries including Australia, Taiwan and United States. We have identified 2 recurrent mutations (*BRCA2* c.3109C>T and *BRCA2* c.4965delC) using the SNaPshot genotyping panel and a novel mutation *BRCA2* c.3165_3167delinsCC by NGS.

To further test the feasibility and reproducibility of the genotyping panel in Chinese emigrants, we additionally collected information of *BRCA1/2* mutation spectrum from overseas collaborating groups. We received data from 84 Chinese breast cancer patients who had known pathogenic *BRCA1/2* mutations, which included 63 different types of mutations (22 *BRCA1* and 41 *BRCA2*). Consistent with our local data, *BRCA2* mutation predominance was seen in overseas Chinese breast cancer patients. As shown in Table 5, only 14 types of mutations were commonly seen in Hong Kong and overseas. Our SNaPshot genotyping panel covered 9 types of these mutations (3 *BRCA1* and 6 *BRCA2)* and should be able to pick up 17 out of the 84 carriers. The most predominant mutations in this overseas spectrum were *BRCA2* c.5164_5165delAG (n=4), c.7379_7382delACAA (n=4) and c.7878G>A (n=4).

**Table 5:** *BRCA1/2* mutations that seen in both Hong Kong and overseas spectrums

Gene	Mutation (HGVS)	Amino acid change	Covered by SNaPshot genotyping panel
*BRCA1*	c.470_471delCT	p.Ser157^*^	Yes
	c.3607C>T	p.Arg1203^*^	No
	c.4148C>G	p.Ser1383^*^	Yes
*BRCA2*	c.755delA	p.Asp252Alafs^*^25	No
	c.2808_2811delACAA	p.Ala938Profs^*^21	Yes
	c.5164_5165delAG	p.Ser1722Tyrfs^*^4	Yes
	c.5238dupT	p.Asn1747^*^	No
	c.7007G>T	p.Gly2313Alafs^*^31	Yes
	c.7133C>G	p.Ser2378^*^	No
	c.7409dupT	p.Thr2471Hisfs^*^4	No
	c.7558C>T	p.Arg2520^*^	No
	c.7878G>A	p.Trp2626^*^	Yes
	c.9097dupA	p.Thr3033Asnfs^*^11	Yes
	c.9393delC	p.Lys3132Asnfs^*^31	No

Note: *BRCA1* c.5470_5477del8 and *BRCA2* c.4965delC were not seen in the Hong Kong cohort, but were covered by the SNaPshot genotyping panel.

## DISCUSSION

The genetic diagnosis of inherited breast cancer is clinically important in terms of disease management, and offering of genetic counseling and preventive strategies for family members who are at risk. The spectrum of *BRCA* mutation varies across countries and ethnicities, and the prevalence and dominance of *BRCA* mutation also varies among different populations [14, 15]. Even in Mainland China, there are more than 55 ethnic groups, in which Han, Manchu and Uyghur have the largest populations. Few studies have identified *BRCA* mutations in Chinese breast cancer patients, and the prevalence was lower than in the West [16, 17]. Identification of Chinese-specific *BRCA1/2* founder and recurrent mutations would be beneficial, not only to the Hong Kong patients of whom the majority are ethnically Chinese, but also to the enormous population in Mainland China and those emigrants in Western countries. The genetic testing panel was designed specifically for Chinese population to achieve better risk assessment and preventive measures [18].

It has been reported that *BRCA1* c.5589del8 and *BRCA2* c.3109C>T are the most common recurrent mutations in Han Chinese [19], in which *BRCA2* c.3109C>T has been confirmed to be a founder mutation in Southern Chinese [13]. It is of great importance to identify the prevalence of *BRCA1/2* or other high-penetrance genes in Chinese population, so that better risk assessment and clinical management can be offered to this subset of the population. In view of this, our pilot study was carried out to assess the prevalence of germline mutation in hereditary breast cancers in Hong Kong. Our previous results showed that less than 10% of the clinically high risk breast cancer patients harbor *BRCA1* and *BRCA2* mutations, and importantly, nearly half of the mutations were recurrent mutations [2, 13]. In consistent with our previous findings, the current mutational screening using the SNaPshot genotyping panel was able to pick up 3.52% (21/596) of *BRCA* carriers among the local cohorts, which covered 43.75% (21/48) of the identified mutations. Furthermore, *BRCA1* c.964delG (n=3), c.4372C>T (n=3) and *BRCA2* c.3109C>T (n=7) were the most common mutations identified by the SNaPshot panel in this study cohort (Table 4). Besides, there were 27 mutation carriers identified by NGS full gene sequencing or MLPA. Of these, most of them were novel to our *BRCA* mutation spectrum, and *BRCA1* c.2635G>T (n=2) and *BRCA2* c.7133C>G (n=3) were newly identified recurrent mutations in Hong Kong cohort. It is expected that more recurrent *BRCA1/2* mutations will be identified as the cohort size increases, understanding the *BRCA* mutation spectrum is important to update and improve the coverage of the SNaPshot panel.

One of the limitations of the SNaPshot genotyping panel and amplicon-based NGS is that large genomic rearrangements in *BRCA1/2* genes could be not detected, which were reported to comprise up to 7% of all *BRCA* mutations in Asians [20]. Therefore, the incorporation of MLPA into *BRCA1/2* testing pipeline is necessary. In this study, one *BRCA1* large deletion (r.5194_5406 del) was identified from local breast cancer patient by this method (Table 5) with a frequency of 2.08% (1/48) among all mutations. Notably, our previous study of a 1,236 high-risk Chinese HBOC patient cohort also showed 8 out of 120 identified mutations (6.67%) were large insertions/deletions [21].

By comparing the mutation spectra with overseas countries, *BRCA2* predominance is common among Chinese patients, which is concordant with the prevalence observed in the local cohort and other studies [19]. The *BRCA1* c.5470_5477delATTGGGCA and *BRCA2* c.7878G>A were the most prevalent mutations among Chinese breast cancer patients that reside in US and other countries. The frequencies of *BRCA* mutations were 10.5% from a cohort of 409 Chinese breast cancer patients in Northern China, and *BRCA2* mutations were more common than *BRCA1* [22]. Large-scale population screening is warranted to unravel the frequencies and penetrance of *BRCA* mutations in Chinese population.

Identification and molecular diagnosis of HBOC families allow healthcare providers and professionals to decide on the clinical management guidelines and strategies. Substantial data from the West pointed to the importance of mutation screening and counselling [23], however, access to genetic services including genetic counselling in Mainland China is still inadequate. The cost of genetic testing and lack of coverage by local healthcare system are the main obstacles for mutation screening in Hong Kong and many parts of Asia [14]. Although next-generation sequencing is getting popular in the field of mutation screening, its high setup costs and the requirements of bioinformatics pipeline and sequencing data analysis are still challenging. Therefore, development of a genotyping panel for targeted recurrent mutation screening can offer a simple, rapid and affordable routine molecular diagnostic method that is feasible in most diagnostic laboratories. In our genetic testing workflow, initial screening using SNaPshot genotyping panel costs about USD 50 per sample with a turn-around time of 3 days, while the *BRCA1/2* full-gene sequencing with NGS cost about USD 1,500 per sample.

Data from this present study demonstrated around 10% of Chinese HBOC families had *BRCA* mutations which emphasizes the significant impact on breast cancer and ovarian cancer risk in the local population. Implementation of the SNaPshot genotyping panel in the mutation screening pipeline is feasible which helps to identify patients who are at risk, and may change the paradigm for treatment in this subset of patients who carry *BRCA* mutation.

## MATERIALS AND METHODS

### Patients and DNA samples

441 high-risk breast cancer probands and 155 unselected ovarian cancer probands were recruited by Hong Kong Hereditary and High Risk Breast Cancer Programme (HRBCP) in Queen Mary Hospital and Tung Wah Hospital in Hong Kong. In addition, 79 overseas Chinese with breast and/or ovarian cancers were recruited from Australia, Taiwan and the United States. Written informed consent was obtained from all participating patients, and this study was approved by the Institutional Review Board (IRB) of The University of Hong Kong, and Hospital Authority Hong Kong West Cluster. Patient information including age, family history, and clinical reports were recorded (Tables 2 and 3). Genomic DNA was extracted from peripheral blood for *BRCA1/2* genetic testing using QIAamp DNA Blood Mini Kit (Qiagen, Hilden, Germany) according to the manufacturer's instructions.

### Genetic testing selection criteria

High-risk breast cancer patients were recruited if any of the following criteria were met: (1) diagnosis of breast cancer at age ≤ 45 years; (2) bilateral breast cancer; (3) triple-negative or medullary type pathology; (4) male breast cancer; (5) at least one first- or second- degree relative with breast and/or ovarian cancer, regardless of age. In the unselected cohort of ovarian cancer, patients who had ovarian, fallopian tube or peritoneal cancer at any age were recruited.

### Genetic testing workflow

Recruited breast and ovarian cancer probands were first tested for the 25 targeted *BRCA1/2* recurrent mutations using the SNaPshot genotyping panel (Table 1). Those who tested negative were assayed for large genomic rearrangements by MLPA and amplicon-based NGS targeting all *BRCA1/2* exons [2]. All mutations identified by the SNaPshot genotyping panel and NGS were further verified by independent Sanger sequencing. The genetic testing workflow was illustrated in Figure 1.

### Mutation screening by single base extension (SBE) assay (SNaPshot)

Twenty-five *BRCA1/2* mutations, including founder and recurrent mutations reported in Chinese ethnic groups, were identified by our group [13] or from literature search [12, 22, 24, 25] (Table 1). These mutations were reported in China, Hong Kong, Malaysia and Singapore Chinese populations, and were all small insertion/deletion or nonsense mutations. Primers designed for PCR amplification and SNaPshot genotyping assays (Applied Biosystems, Thermo Fisher Scientific, MA, USA) were listed in Supplementary Tables 1 and 2. The single base extension assay, SNaPshot, has been adopted for mutation screening. In brief, all 25 mutation loci were amplified by multiplex PCR. The mutations were examined by primers positioned immediately 5′ to the heterozygous bases. Single base extension reaction was performed in multiplex using the SNaPshot kit and modified primers with linkers of different repeating units of oligo TGAC. After purification, the SNaPshot products were run with 120 LIZ size standard in sequencing analyzer e.g. ABI Prism 3730. Data were processed by Genescan Analysis (Applied Biosystems). Relative intensity of the two alleles in each sample was calculated and confirmed the heterozygosity. The signal from the normal control DNA was used as reference.

### Next-generation sequencing (NGS)

Samples tested negative from the SNaPshot genotyping panel were further assayed for *BRCA1/2* full-gene sequencing by NGS. The Fluidigm Access Array System (Fluidigm, San Francisco, USA) was used to generate separate pools of 74 PCR amplicons per sample for targeting all exons of the *BRCA* genes plus 10 bp from intron-exon boundaries as previously described [26]. 768 barcode combinations were used in rotation to minimize using the same combination in two consecutive runs. Paired-end sequencing of the amplicons (2 × 300bp) was performed on a MiSeq (Illumina, San Diego, USA) with reagent kit v3.

Sequences obtained from each sample were mapped to human genome reference GRCh37/hg19 using BWA-MEM version 0.7.7 with default parameters [27]. The minimum sequencing depth of coverage for ROI was 100x. Two variant callers were used: (1) Samtools version 0.1.9 [28], with mpileup command parameters -L 100000 -d 100000 to cater for amplicons with depth exceeding 250-fold and bcftools command parameter –m0.99 to use the new insertion-deletion (INDEL) calling model; (2) GATK HaplotypeCaller version 2.8-1 [29] according to the best practices as mentioned previously [2]. Variant calls were annotated by Ensembl Variant Effect Predictor version 75 [30]. The bioinformatic analysis was performed on a Cray XC30 supercomputer (Cray, Seattle, USA). Pathogenic mutations or ROI positions with depth of coverage below 100x were subject to Sanger sequencing and data analysis by Mutation Surveyor version 4.0.6 (Softgenetics Inc, USA).

### *BRCA* mutation validation by DNA sequencing

All *BRCA* mutations identified from SNaPshot genotyping panel and NGS were further validated by Sanger sequencing. Sequencing results were compared with the reference DNA sequences using Variant Reporter software (Applied Biosystems) and then reviewed manually. Computational analysis for potential cryptic splice site mutations were performed using splice site prediction programs such as NNSPLICE and ESEF finder. All mutations were named according to the recommendations from Human Genome Variation Society (HGVS). Variant descriptions were checked by Mutalyzer Name Checker (http://mutalyzer.nl). DNA sequencing was supplemented by multiplex ligation dependent probe amplification (MLPA) to detect large deletions or rearrangements [21, 31].

## SUPPLEMENTARY MATERIALS TABLES



## References

[1] Hong Kong Cancer Registry Hospital Authority. Female Breast Cancer in 2014.

[2] Kwong A, Shin VY, Au CH, Law FB, Ho DN, Ip BK, Wong AT, Lau SS, To RM, Choy G, Ford JM, Ma ES, Chan TL (2016). Detection of germline mutation in hereditary breast and/or ovarian cancers by next-generation sequencing on a four-gene panel. J Mol Diagn.

[3] Hartmann LC, Sellers TA, Schaid DJ, Frank TS, Soderberg CL, Sitta DL, Frost MH, Grant CS, Donohue JH, Woods JE, McDonnell SK, Vockley CW, Deffenbaugh A (2001). Efficacy of bilateral prophylactic mastectomy in BRCA1 and BRCA2 gene mutation carriers. J Natl Cancer Inst.

[4] Domchek SM, Weber BL (2006). Clinical management of BRCA1 and BRCA2 mutation carriers. Oncogene.

[5] Rebbeck TR, Lynch HT, Neuhausen SL, Narod SA, Van't Veer L, Garber JE, Evans G, Isaacs C, Daly MB, Matloff E, Olopade OI, Weber BL, Prevention and Observation of Surgical End Points Study Group (2002). Prophylactic oophorectomy in carriers of BRCA1 or BRCA2 mutations. N Engl J Med.

[6] Isakoff SJ, Mayer EL, He L, Traina TA, Carey LA, Krag KJ, Rugo HS, Liu MC, Stearns V, Come SE, Timms KM, Hartman AR, Borger DR (2015). TBCRC009: a multicenter phase II clinical trial of platinum monotherapy with biomarker assessment in metastatic triple-negative breast cancer. J Clin Oncol.

[7] Ledermann J, Harter P, Gourley C, Friedlander M, Vergote I, Rustin G, Scott CL, Meier W, Shapira-Frommer R, Safra T, Matei D, Fielding A, Spencer S (2014). Olaparib maintenance therapy in patients with platinum-sensitive relapsed serous ovarian cancer: a preplanned retrospective analysis of outcomes by BRCA status in a randomised phase 2 trial. Lancet Oncol.

[8] Levy-Lahad E, Catane R, Eisenberg S, Kaufman B, Hornreich G, Lishinsky E, Shohat M, Weber BL, Beller U, Lahad A, Halle D (1997). Founder BRCA1 and BRCA2 mutations in Ashkenazi Jews in Israel: frequency and differential penetrance in ovarian cancer and in breast-ovarian cancer families. Am J Hum Genet.

[9] Roa BB, Boyd AA, Volcik K, Richards CS (1996). Ashkenazi Jewish population frequencies for common mutations in BRCA1 and BRCA2. Nat Genet.

[10] Janavicius R (2010). Founder BRCA1/2 mutations in the Europe: implications for hereditary breast-ovarian cancer prevention and control. EPMA J.

[11] Chen W, Zheng R, Baade PD, Zhang S, Zeng H, Bray F, Jemal A, Yu XQ, He J (2016). Cancer statistics in China, 2015. CA Cancer J Clin.

[12] Li WF, Hu Z, Rao NY, Song CG, Zhang B, Cao MZ, Su FX, Wang YS, He PQ, Di GH, Shen KW, Wu J, Lu JS (2008). The prevalence of BRCA1 and BRCA2 germline mutations in high-risk breast cancer patients of Chinese Han nationality: two recurrent mutations were identified. Breast Cancer Res Treat.

[13] Kwong A, Ng EK, Wong CL, Law FB, Au T, Wong HN, Kurian AW, West DW, Ford JM, Ma ES (2012). Identification of BRCA1/2 founder mutations in Southern Chinese breast cancer patients using gene sequencing and high resolution DNA melting analysis. PLoS One.

[14] Nakamura S, Kwong A, Kim SW, Iau P, Patmasiriwat P, Dofitas R, Aryandono T, Hu Z, Huang CS, Ginsburg O, Rashid MU, Sarin R, Teo SH (2016). Current status of the management of hereditary breast and ovarian cancer in Asia: first report by the Asian BRCA consortium. Public Health Genomics.

[15] Alemar B, Herzog J, Brinckmann Oliveira Netto C, Artigalas O, Schwartz IV, Matzenbacher Bittar C, Ashton-Prolla P, Weitzel JN (2016). Prevalence of Hispanic BRCA1 and BRCA2 mutations among hereditary breast and ovarian cancer patients from Brazil reveals differences among Latin American populations. Cancer Genet.

[16] Li SS, Tseng HM, Yang TP, Liu CH, Teng SJ, Huang HW, Chen LM, Kao HW, Chen JH, Tseng JN, Chen A, Hou MF, Huang TJ (1999). Molecular characterization of germline mutations in the BRCA1 and BRCA2 genes from breast cancer families in Taiwan. Hum Genet.

[17] Song CG, Hu Z, Wu J, Luo JM, Shen ZZ, Huang W, Shao ZM (2006). The prevalence of BRCA1 and BRCA2 mutations in eastern Chinese women with breast cancer. J Cancer Res Clin Oncol.

[18] Li T, Mello-Thoms C, Brennan PC (2016). Descriptive epidemiology of breast cancer in China: incidence, mortality, survival and prevalence. Breast Cancer Res Treat.

[19] Cao W, Wang X, Li JC (2013). Hereditary breast cancer in the Han Chinese population. J Epidemiol.

[20] Kim H, Choi DH (2013). Distribution of BRCA1 and BRCA2 mutations in Asian patients with breast cancer. J Breast Cancer.

[21] Kwong A, Chen J, Shin VY, Ho JC, Law FB, Au CH, Chan TL, Ma ES, Ford JM (2015). The importance of analysis of long-range rearrangement of BRCA1 and BRCA2 in genetic diagnosis of familial breast cancer. Cancer Genet.

[22] Zhang J, Pei R, Pang Z, Ouyang T, Li J, Wang T, Fan Z, Fan T, Lin B, Xie Y (2012). Prevalence and characterization of BRCA1 and BRCA2 germline mutations in Chinese women with familial breast cancer. Breast Cancer Res Treat.

[23] Desmond A, Kurian AW, Gabree M, Mills MA, Anderson MJ, Kobayashi Y, Horick N, Yang S, Shannon KM, Tung N, Ford JM, Lincoln SE, Ellisen LW (2015). Clinical actionability of multigene panel testing for hereditary breast and ovarian cancer risk assessment. JAMA Oncol.

[24] Khoo US, Chan KY, Cheung AN, Xue WC, Shen DH, Fung KY, Ngan HY, Choy KW, Pang CP, Poon CS, Poon AY, Ozcelik H (2002). Recurrent BRCA1 and BRCA2 germline mutations in ovarian cancer: a founder mutation of BRCA1 identified in the Chinese population. Hum Mutat.

[25] Kurian AW, Gong GD, Chun NM, Mills MA, Staton AD, Kingham KE, Crawford BB, Lee R, Chan S, Donlon SS, Ridge Y, Panabaker K, West DW (2008). Performance of BRCA1/2 mutation prediction models in Asian Americans. J Clin Oncol.

[26] Kwong A, Shin VY, Cheuk IW, Chen J, Au CH, Ho DN, Chan TL, Ma ES, Akbari MR, Narod SA (2016). Germline RECQL mutations in high risk Chinese breast cancer patients. Breast Cancer Res Treat.

[27] Li H (2014). Toward better understanding of artifacts in variant calling from high-coverage samples. Bioinformatics.

[28] Li H, Handsaker B, Wysoker A, Fennell T, Ruan J, Homer N, Marth G, Abecasis G, Durbin R, Genome Project Data Processing Subgroup (2009). The Sequence Alignment/Map format and SAMtools. Bioinformatics.

[29] DePristo MA, Banks E, Poplin R, Garimella KV, Maguire JR, Hartl C, Philippakis AA, del Angel G, Rivas MA, Hanna M, McKenna A, Fennell TJ, Kernytsky AM (2011). A framework for variation discovery and genotyping using next-generation DNA sequencing data. Nat Genet.

[30] McLaren W, Pritchard B, Rios D, Chen Y, Flicek P, Cunningham F (2010). Deriving the consequences of genomic variants with the Ensembl API and SNP Effect Predictor. Bioinformatics.

[31] Kwong A, Ng EK, Law FB, Wong HN, Wa A, Wong CL, Kurian AW, West DW, Ford JM, Ma ES (2012). Novel BRCA1 and BRCA2 genomic rearrangements in Southern Chinese breast/ovarian cancer patients. Breast Cancer Res Treat.

