# Supplementary material for: Rapid detection of BRCA1/2 recurrent mutations in Chinese breast and ovarian cancer patients with multiplex SNaPshot genotyping panels
Source: Oncotarget. 2017 Dec 20;9(8):7832–43. doi: 10.18632/oncotarget.23471 (PMC5814262; doi:10.18632/oncotarget.23471)
Supplement: Supplementary file 1 [file oncotarget-09-7832-s001.pdf]

Rapid detection of *BRCA1/2* recurrent mutations in Chinese breast and ovarian cancer patients with multiplex SNaPshot genotyping panels

SUPPLEMENTARY MATERIALS

Supplementary Table 1: Primer sequences used for PCR amplification of the 25 *BRCA1/2* recurrent mutation loci

Nucleotide change	Forward primer sequence (5'-3')	Reverse primer sequence (5'-3')	Amplicon size (bp)	Multiplex PCR *
BRCA1				
c.470_471delCT	TGTTAGCTGACTGATGATGGT	ATCCAGCAATTATTATTAAATAC	267	B
c.964delG	TTACTCACTAAAGACAGAATGA	CCAGAATATTCATCTACCTCA	401	F
c.981_982delAT	TTACTCACTAAAGACAGAATGA	CCAGAATATTCATCTACCTCA	401	B
c.2253_2254delGT	ACAGCAGAAACCTACAACTC	AAGCCTTCTGTGTCATTTCT	503	C
c.3333delA	CAAAATTGAATGCTATGCTTAGA	TCGGTAACCCTGAGCCAAAT	377	G
c.3342_3345delAGAA	CAAAATTGAATGCTATGCTTAGA	TCGGTAACCCTGAGCCAAAT	377	D
c.3916_3917delTT	GTAATATTGGCAAAGGCATCT	TAAAATGTGCTCCCCAAAAGCA	360	E
c.4065_4068delTCAA	GTAATATTGGCAAAGGCATCT	TAAAATGTGCTCCCCAAAAGCA	360	I
c.4148C>G	GTCCTGCCAATGAGAAGAAA	TGTCAGCAAACCTAAGAATGT	265	D
c.4372C>T	CTAACCTGAATTATCACTATCA	GTGTATAAATGCCTGTATGCA	312	E
c.5406+1_5406+3delGTA	TCCCATTGAGAGGTCTTGCT	GAGAAGACTTCTGAGGCTAC	297	D
c.5406+7A>G	TCCCATTGAGAGGTCTTGCT	GAGAAGACTTCTGAGGCTAC	297	I
c.5470_5477del ATTGGGCA	ATGAATTGACACTAATCTCTGC	GTAGCCAGGACAGTAGAAGGA	280	C
BRCA2				
c.1832C>A	CAGCATCTTGAATCTCATACAG	CATGTATACAGATGATGCCTAAG	625	F
c.2595delA	ATGGAAAAGAATCAAGATGTAT	CTTAATGTTATGTTCAGAGAG	633	G
c.2808_2811delACAA	ATGGAAAAGAATCAAGATGTAT	CTTAATGTTATGTTCAGAGAG	633	E
c.3109C>T	TTTGGAGGTAGCTTCAGAAC	TTCTGCAATATGTAGCTTGG	404	B
c.4965delC	CAGCTAGCGGGAAAAAAGTTA	TTCGGAGAGATGATTTTTGTC	626	A
c.5164_5165delAG	AAAAATGGCTTAGAGAAGGAA	ATCCTCTGAATCATCCAATG	638	A
c.6591_6592delTG	CCAGAGCACTGTGTAAACTC	TACTCCCCCAAACTGACTAC	641	F
c.7007G>T	TTTATGCTGATTTCTGTTGTAT	ACGAGACTTTTCTCATACTGT	349	H
c.7878G>A	CAGAGAATAGTTGTAGTTGTT	AACCTTAACCATACTGCC	303	G
c.8068_8069delGT	TTTTATTCTCAGTTATTCAGTG	GAAATTGAGCATCCTTAGTAA	546	H
c.9097dupA	ATCACTTCTTCCATTGCATC	CCGTGGCTGGTAAATCTG	338	C
c.9294C>G	CTATTTTGATTTGCTTTTATTATT	GCTATTTCCTTGATACTGGAC	447	H

* 9 designated sets of multiplex PCR (A to I).

Supplementary Table 2: Detection oligo sequences for multiplex SNaPshot reactions

Gene	Detected mutation	Extension primer sequence (5'-3')	Length (nt)	Base change
Panel 1				
BRCA1	c.981_982delAT	GGAGTCCGCCTATCATTAC	19	A/G
BRCA1	c.2253_2254delGT	CTGACTGACTGACTGAAGACCCCAAAGATCTCAT	34	G/T
BRCA1	c.5470_5477delATTGGGCA	ACTGACTGACTGACTGACTTGTGTTCTCTGTCTCCAGCA	39	A/G
BRCA2	c.3109C>T	GACTGACTGACTGACTGACTCACAAGCTAAACTAGTAGGATATT	44	G/A
BRCA2	c.4965delC	TGACTGACTGACTGACTGACTGACTGACTGAATAAGGGGACTGATTTGT	49	G/T
BRCA2	c.5164_5165delAG	ACTGACTGACTGACTGACTGACTGACTGACTTTTTGTCATTTTCAGCTATAGTA	54	C/G
BRCA2	c.9097dupA	ACTGACTGACTGACTGACTGACTGACTGACTGACTGACTGTTAGCAGCGACAAAAAAAA	59	C/A
BRCA1	c.470_471delCT	GACTGACTGACTGACTGACTGACTGACTGACTGACTGACTGACTGACTCCAGTCTCAGTGTCCAACTCT	69	C/A
Panel 2				
BRCA1	c.4148C>G	ACTCTGAGAGGATAGCCCT	19	G/C
BRCA1	c.3916_3917delTT	GACTAGTGCAGTGAATTGGAAGAC	24	T/G
BRCA1	c.4372C>T	CTGACTGACTCCATTTAAAGCAGTATTAACTTCA	34	C/T
BRCA1	c.5406+1_5406+3delGTA	GACTGACTGACTGACTGACTGACAGGGCACCCAATACTT	39	A/G
BRCA2	c.1832C>A	GACTGACTGACTGACTGACTGACTAATACCGAAAGACCAAAAAT	44	C/A
BRCA2	c.2808_2811delACAA	CTGACTGACTGACTGACTGACTGACTTTATATGGAGACACAGGTGATAA	49	A/G
BRCA1	c.964delG	CTGACTGACTGACTGACTGACTGACTGACTGACTATGTTTCCTTACTTCCAGCC	54	C/A
BRCA2	c.6591_6592delTG	TGACTGACTGACTGACTGACTGACTGACTGACTGACTACAGGAACATCAGAAAAAGTTT	59	C/G
BRCA1	c.3342_3345delAGAA	GACTGACTGACTGACTGACTGACTGACTGACTGACTGACTGACTATCTGTATTAACAGTCTGAACTACT	69	T/C
Panel 3				
BRCA2	c.7007G>T	CGATTACCTGTGTACCCTTTC	21	G/T
BRCA1	c.3333delA	CTAGTCTGAACTACTTCTTCATATTCT	27	T/G
BRCA2	c.2595delA	TGACTGACTGACTAGAGTAATCCAAAAAAATCAAGA	36	A/G
BRCA2	c.7878G>A	ACTGACTGACTGACTTTGGGTTTATAATCACTATAGATG	39	G/A
BRCA2	c.9294C>G	CTGACTGACTGACTGACTGACTGTCTATTTGTCAGACGAATGTTA	45	C/G
BRCA2	c.8068_8069delGT	GACTGACTGACTGACTGACTGACTGACTTCAATGAAATTATGTCAGAAACA	51	C/G
BRCA1	c.5406+7A>G	ACTGACTGACTGACTGACTGACTGACTGACTGACTGACTCTCTGACAGGGCACCCAA	57	T/C
BRCA1	c.4065_4068delTCAA	TGACTGACTGACTGACTGACTGACTGACTGACTGACTGACTGACTGGGCTTGGAAGAAAATAA	63	T/G
